# Water Sustainability at the River Grande Basin, Brazil: An Approach Based on the Barometer of Sustainability

**DOI:** 10.3390/ijerph15112582

**Published:** 2018-11-19

**Authors:** Janaína Ferreira Guidolini, Angélica Giarolla, Peter Mann Toledo, Carlos Alberto Valera, Jean Pierre Henry Balbaud Ometto

**Affiliations:** 1 National Institute for Space Research (INPE), Earth System Science Center, São José dos Campos 12227010, SP, Brazil; angelica.giarolla@inpe.br (A.G); peter.toledo@inpe.br (P.M.T); jean.ometto@inpe.br (J.P.H.B.O); 2 Public Ministry of the State of Minas Gerais, Uberaba 38010140, MG, Brazil; carlosvalera@mpmg.mp.br

**Keywords:** water management, sustainable development, water governance, health environment, human well-being

## Abstract

Water resources are fundamental for the social and economic development of a country and sustainability is the best approach to treat water-related problems. Therefore, sustainability studies of water resources are deemed urgent. Sustainability analysis methods should enable space-temporal monitoring, decision-making, and development of policies necessary for water governance. Furthermore, sustainability analysis methods should also integrate environment and socioeconomic variables into a single system. In this context, this study aimed to assess the water sustainability conditions of the River Grande Basin (BHRG), Brazil, before the implementation of the Integrated Water Resources Plan (IWRP), using the Barometer of Sustainability tool (BS). The River Grande basin was in an “almost unsustainable” condition and under high environmental stress. A significant imbalance between environmental and human well-being in the system was also observed. To achieve an acceptable sustainability condition, it is thus necessary to improve the environmental quality of the area. Among the priority thematic area, native vegetation recovery was the most urgent. Overall, the sustainability study based on the BS not only facilitates comprehension regarding environment and human interrelationships, but also provide references for policy formulations and water management.

## 1. Introduction

A sustainable future for new generations is a fundamental goal for natural resource planning and management, and one of the biggest challenges of the 21st century [1,2]. A sustainable future requires a development that “meets the needs of the present without compromising the ability of future generations to meet their own needs” [3] (p. 16). Sustainability comprises “three pillars” or “three dimensions”—environment, economy, and society. However, their interrelationships, particularly regarding the degree of substitutability between natural and human capital, have been at the center of the debate on “weak sustainability” versus “strong sustainability” [4]. For weak sustainability, capital grows almost in an unlimited manner, basically because it believes that produced capital and natural capital can easily replace each other. However, strong sustainability considers a limited substitutability between produced capital and natural capital [5].

The traditional water management concept does not meet management requirements for sustainable development, as it considers water resources a public administration “sector”. Moreover, it believes that water is antagonistic to the sectors that use water resources, such as agriculture, industry, transportation, basic sanitation, and electricity generation. Due to its multiple uses, water transposes all these specialized sectors, in addition to being fundamental to the local population of the region where it is located. Integrated water management aims to ensure water resource preservation, use, recovery, and conservation in conditions satisfactory to their multiple users and consistent with the region’s balanced and sustainable development and efficiency [6]. The water resources area requires managers to make decisions under high system complexities and uncertainties, which demand the integration of information relevant to the issue [7]. Thus, researchers and policy-makers defend sustainability as the best approach to current and future water-related problems. For this approach, the integration between ecosystem conservation and social, economic, and institutional aspects into a single system is necessary [8]. River basins are a natural unit, or a unique system, that allows for this integration [9,10].

Measuring sustainability is an interdisciplinary and complex work [10]. Indicators are powerful tools for assessing progress towards sustainable development [11]. Working with indicators is strongly recommended by researchers [12,13,14], decision-makers [15], and international institutions [16,17]. The use of water-related indicators can contribute not only to improving water allocation [11] and management [10], but also in subsidizing public policy formulations [2]. Nonetheless, several possible difficulties in working with indicators are noted, as work scale, data availability, frequency, and data collection feasibility, among others, can compromise sustainability analyses.

Thus, sustainability analysis methodologies should be flexible (especially with regard to indicator choice), making it possible to easily apply them at different work scales with a didactic presentation of the results. Water resource sustainability analysis methods, besides enabling monitoring, should also provide information that can guide decision-making and policy development necessary for community and regional water governance. Furthermore, they should also increase sustainability awareness through social learning (increasing society participation and protection) and be useful for scientific research and sustainability analyses [1]. The water resources issue is fundamental for the social and economic development of a country [18]. Therefore, sustainability studies of water resources are deemed urgent. The authors of [19] assessed the main sustainability analysis methods in their studies, namely Ecological Footprint (1993), Barometer of Sustainability (1997), and Dashboard of Sustainability (1999). These techniques are advantageous, as they are easily applied and understood, aid in decision-making, and provide specific information on sustainability conditions. Nevertheless, the authors point out the disadvantages of each method. The Ecological Footprint is a static method and does not approach the social and economic dimensions, while the Barometer of Sustainability can be subjective during the performance scale elaboration process. The Dashboard of Sustainability is complex, concerning the scope of interaction between the economic, social, environmental, and institutional dimensions. As for comprehensive water resources indexes, the most common approaches are the Water Poverty Index (WPI), the Integrated Water Stress Index, the Water Scarcity or Shortage Index, the Water Resources Carrying Capacity Index, the Water Security Index, and the Human-Water Harmony Index. Both constitute a system of fixed indicators [20].

The Barometer of Sustainability (BS) can be used as a communication tool, focusing discussion on the meaning of human well-being and ecosystem well-being, their inter-relationships and the importance of both for sustainable development. The differences and similarities between population perception and conventional data will soon become apparent. This can then act as a focus of discussion among managers, scientists, and development workers to arrive at a common understanding of the problems of this area [21] One of the advantages of the system is its holistic approach, achieved through human well-being with the environment. Human well-being and the environment are combined in an appropriate manner, seeking to preserve the process information. The decline of one index does not mask the growth of another; this is particularly important in the general index [22]. Calculations are somewhat complex and can only be performed if certain numerical goals or standards exist. The system uses a percentage scale to measure this performance, by applying human and ecosystem well-being indices, calculating the sub-indices, and providing comparative data and graphical presentation devices [23]. The performance scale is considered extremely subjective by many authors [22]. However, this type of scale is no more or less subjective than any other monetization method currently in use. In addition, the major advantage of this tool is the transparency of the scale of performance when compared to other methods. In BS, performance scale measures considered good or unacceptable should be explicitly defined [21]. Moreover, the BS can be applied from a local to global scale, allowing for spatio-temporal monitoring and comparisons, subsidizing decision-making based on the identification of priority areas [24,25,26,27,28], and facilitating knowledge on the relationships between humans and environment [22]. Although is feasible at the most diverse scales, there are few sustainability evaluations in watersheds in the literature using the BS tool. The BS tool was applied at the Jurumirim watershed, in Rio de Janeiro, Brazil [24]. In Brazil, BS is applied at municipal and state scales, more frequently [26,27,28].

The National Water Resources Policy [29] provides that the first step in the integrated management of a river basin is to carry out a diagnosis of the water resource situation. This step aids managers in understanding the current conditions of basins and assists in the identification and prioritization of main problems [30]. The diagnosis of the BHRG was elaborated using the DPSIR (Driving Force-Pressure-State-Impact-Response) methodology. This methodology analyzes the water situation and determines the capacity of the analyzed indicators to change the state of the water in the basin [31]. The most serious objection to this approach on indicators is that this methodology neglects the systemic and dynamic nature of the processes that occur in a river basin [23,32]. The DPSIR model is based on chance and considers the effect as a function of the cause. That is, it disregards the interaction between the analyzed variables. Disregarding the interaction between variables can lead to erroneous institutional policies, and very simplified interpretation of the interrelationships between the variables to be measured will be obtained [33,34,35]. The diagnosis of the water resources situation is the main source for the drafting of integrated river basin plans. In this context, this study aimed to assess the water sustainability condition at theBHRG, Brazil, before the implementation of the Integrated Water Resources Plan (IWRP) based on the Barometer of Sustainability (BS) method. Furthermore, we sought not only to understand human and environmental interrelationships, but also determine priority thematic areas, which demand actions and/or urgent intervention by managers and society. This study may aid in the spatial-temporal monitoring of the IWRP, after its implementation, as it applied a system of diagnosis indicators concerning the situation of the BHRG water resources. The results were discussed under the sustainability approach, integrating the analyzed environmental, economic, and social variables.

## 2. Material and Methods

### 2.1. Study Area

**Figure 1:**
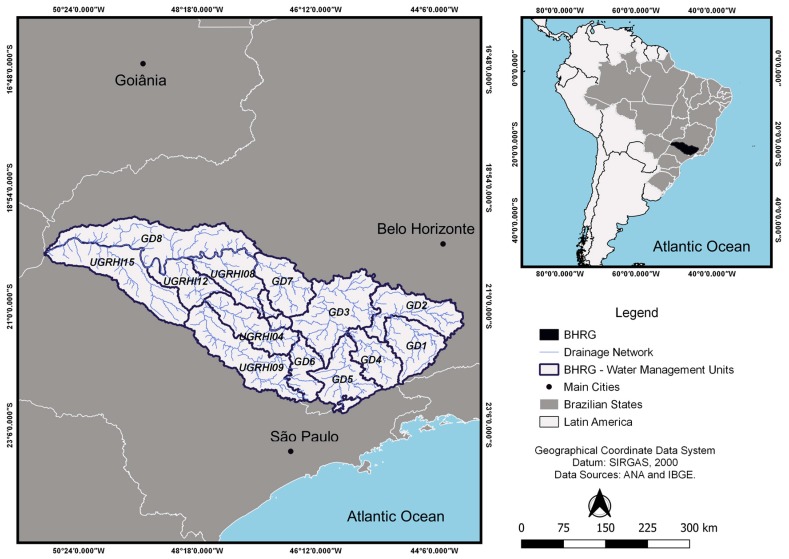
River Grande Basin (BHRG) and its respective Water Management Units (UGHs), located in Minas Gerais State (GDs) and São Paulo State (UGRHIs).

The BHRG is located in southeastern Brazil. The river basin contribution area is of 144,689.54 km², distributed between the states of Minas Gerais (MG)—60% of the total basin area, and São Paulo (SP)—40% of the total basin area. BHRG comprises 393 municipalities, totally or partially inserted in the area, home to 8.6 million people, corresponding to 4.5% of the Brazilian population in 2010 [36]. Inserted in the Paraná Hydrographic Region, one of the most important hydrographic regions in Brazil, the river basin is subdivided into 14 water management units (UGHs). UGHs are administered by state river basin committees and are given different denominations in MG and SP. In MG, the eight UGHs affluent to the Rio Grande are named UPGRHs—Units of Planning and Management of Water Resources, coded “GDs”. In São Paulo, the six UGHs affluent to the Rio Grande are known as “UGRHIs”—Water Resources Management Units (Figure 1).

The BHRG relief is predominantly flat, with some smooth wavy regions. The main types of soil in the region are Ferralsols, Acrisols, and Cambisols. The Rio Grande Basin comprises a rich and diversified biodiversity, with covered by Mata Atlântica and Cerrado biome vegetation. The climate is characterized by rainy summers and dry winters [10].

In geoeconomic terms, BHRG is located in an important region polarized by the axes confluence between the cities of Brasília, Rio de Janeiro, Goiania, Belo Horizonte, and São Paulo, which concentrate the main economic flows of the country. According to the IBGE (Brazilian Institute of Geography and Statistics), the Rio Grande basin region is responsible for one of the largest gross domestic products (GDPs) in Brazil (R$ 189.5 billion in 2011, representing 4.6% of national GDP). Agricultural activities occupy 83% of the total river basin area. The industrial and service sectors in urban centers and hydroelectric power generation are also noteworthy [36].

Most of the recorded water use conflicts at the River Grande Basin relate to environmental sanitation (sewage and solid waste) and diffuse pollution due to agricultural activities and soil erosion [10].

### 2.2. Indicator Selection and Organization into Themes and Sustainable Development Dimensions

**Figure 2:**
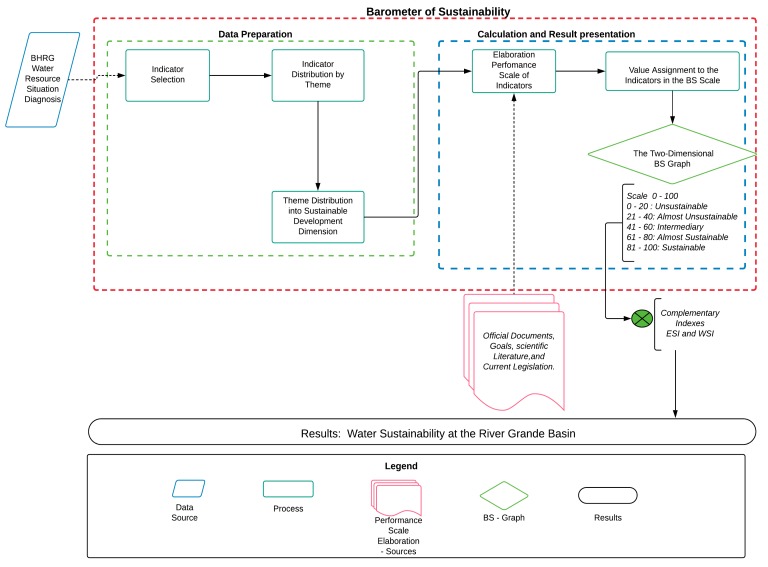
Schematic outline of the present study.

The research design is displayed in Figure 2. First, the sustainability indicators were selected from the Rio Grande Basin Water Resources Situation Diagnosis. This official River Grande Basin document was published in 2008, before the IWRP implementation [31]. Indicator selection was based on the official document because the data were expressed in a watershed scale and separated by UGH, the objects of interest in this study. A total of 22 sustainability indicators were selected, with data availability from the two assessed states (Minas Gerais and São Paulo).

**Figure 3:**
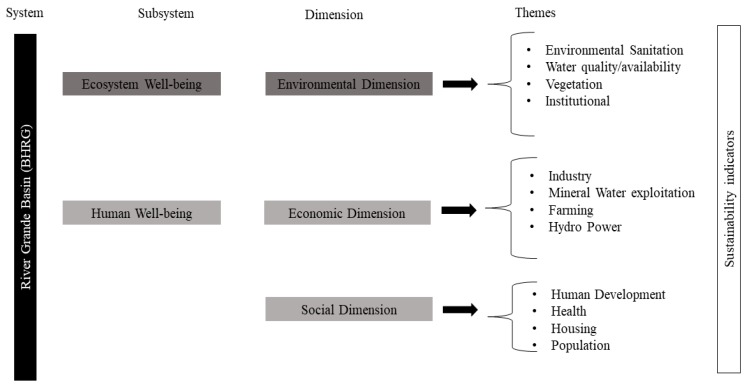
Sustainable development indicators’ organization in each subsystem, dimension, and theme.

After selection, the indicators were distributed by topic and, subsequently, by sustainable development dimensions (environmental, economic, and social dimensions). The subsystem “human well-being” consisted of the social and economic dimensions, while the subsystem “ecosystem well-being” comprised the environmental dimension (Figure 3).

### 2.3. Performance Scale Elaboration to the Selected Indicators

The construction of the performance scale considered the pressure that environmental, social, and economic components exerted on the water resources at BHRG. The BS scale is divided into five ranges, categorized from unsustainable to sustainable. The performance scale works in a similar way. However, the values are represented by the standards established at a local, national, or worldwide level, scientific literature or “goals to be achieved” representation [24]. Thus, it is possible to use the performance scale to evaluate the status of the indicator in relation to the reference (official documents, scientific literature, goals, current legislation) and enable its elaboration for different periods, in order to monitor progress or setbacks towards sustainable development.

References and descriptions of the selected indicators (separated by dimension and theme) are presented Tables S1–S3. The thresholds of the performance scale intervals were defined from the reference value. These thresholds correspond to values ranging from 0 to 100 in the BS scale, pointing to conditions ranging from sustainable (81–100), potentially sustainable (61–80), intermediate (41–60), potentially unsustainable (21–40), and unsustainable (0–20) [21].

Indicators may present an increasing or decreasing scale of performance. For indicators with an increasing performance scale, the highest value point is the best performance. An example of a growing indicator is ‘proportion of the municipalities with landfill’. For indicators with a decreasing performance scale, the lowest value indicates better performance. An example of a decreasing indicator is ‘annual number of hospitalization records for waterborne diseases’.

The actual values of the indicators are shown in Tables S4 and S5 for Minas Gerais and São Paulo, respectively. The indicator performance scales associated with the Sustainability Barometer scale can be observed in Table S6.

### 2.4. Value Assignment to the Indicators in the BS Scale

After the scale performance elaboration, presented in Table S4, the actual value of the indicator was transposed to the BS scale by a simple linear interpolation, followed by the calculation of the arithmetic mean from the lowest to the highest hierarchy level, from the indicator to the theme, from the theme to the dimension and from the dimension to the subsystem (environmental well-being and human well-being). Indicators did not receive any weights, because they were considered equally relevant. The indicator value in the BS scale calculated as:

(1) $$\;\mathrm{BSx}=\lfloor \lfloor \frac{\left(\mathrm{DLa}-\mathrm{DLx}\right)\times \left(\mathrm{BSa}-\mathrm{BSp}\right)}{\left(\mathrm{DLa}-\mathrm{DLp}\right)}\rfloor \times \left(-1\right)\rfloor +\mathrm{BSa}\;$$

where:

BSx = BS scale value.

DLa = previous limit of the range that contains X in the local performance scale.

DLx = actual value of the indicator

BSa = previous limit of the interval that contains X in the barometer scale.

BSp = subsequent limit of the range that contains X in the barometer scale.

DLp = subsequent limit of the range that contains X in the local performance scale.

The sustainability condition of the BHRG water resources and their respective UGHs are presented in a two-dimensional graph, where the X-axis refers to environmental well-being and the Y-axis refers to human well-being [21].

The complementary indexes ESI (Environmental Stress Index) and WSI (Well-being/Stress Index) [16] were calculated from the BS results. These additional evaluation indices attempt to better study the relationship between human well-being and environmental pressure [22].

The ESI represents the residual environmental percentage towards the ideal development situation under sustainability precepts.

The higher this percentage, the higher the stress undergone by the environment. This index is calculated as follows:

(2)  ESI=100−EWI 

where:

EWI = Environmental Well-being Index (calculated from the Sustainability Barometer), comprising the “environmental well-being” subsystem results.

The WSI or Stress/Well-being Index, portrays the cost of human well-being to the environment. The WSI is calculated as follows:

(3)  WSI=HWI÷ESI 

where:

HWI = Human Well-being Index (calculated through the Sustainability Barometer), comprising the “human welfare” subsystem results.

ESI = Environmental Stress Index

High HWI and a WSI close to 1 indicate a high environmental cost for the human well-being maintenance. Low HWI and a WSI close to 1 indicate a socially precarious situation and low environmental exploration. Ideally, the WSI is >1, with high human well-being and low environmental stress. In general, these complementary indices are related to the level of stress suffered by the environment to maintain human well-being.

Finally, the priority areas were calculated through Equation 4. The means of each theme was calculated for Minas Gerais and São Paulo, based on the values obtained in the BS scale, and presented in a hierarchical graph. The highest values (presented in darker tones) represented the most critical thematic areas, i.e., that demand actions and/or urgent intervention by managers and society.

(4)  AP=100−BSMt 

where:

AP = Priority thematic area

BSMt = Mean value of the theme in the BS scale

## 3. Results and Discussion

### 3.1. Water Resources’ Sustainability Condition at the River Grande Basin

**Figure 4:**
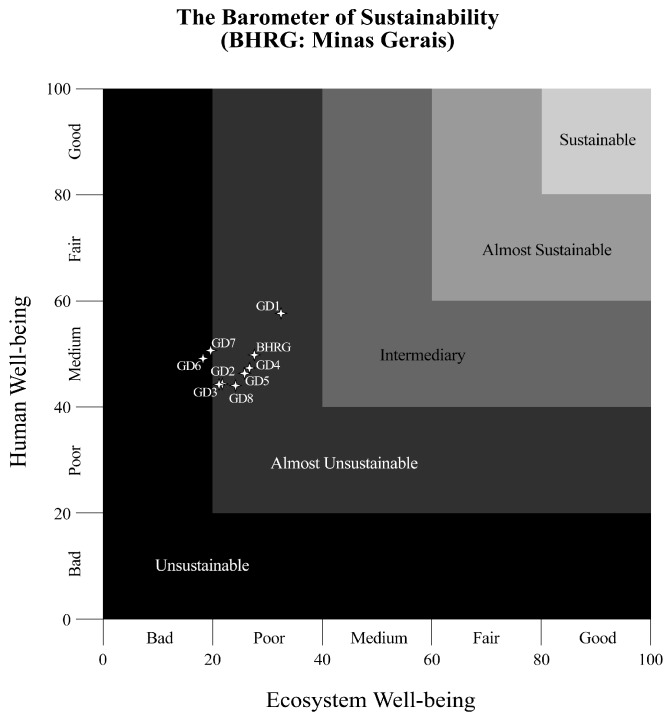
Two-dimensional graphs representing water sustainability condition at BHRG and its respective water management units in Minas Gerais.

**Figure 5:**
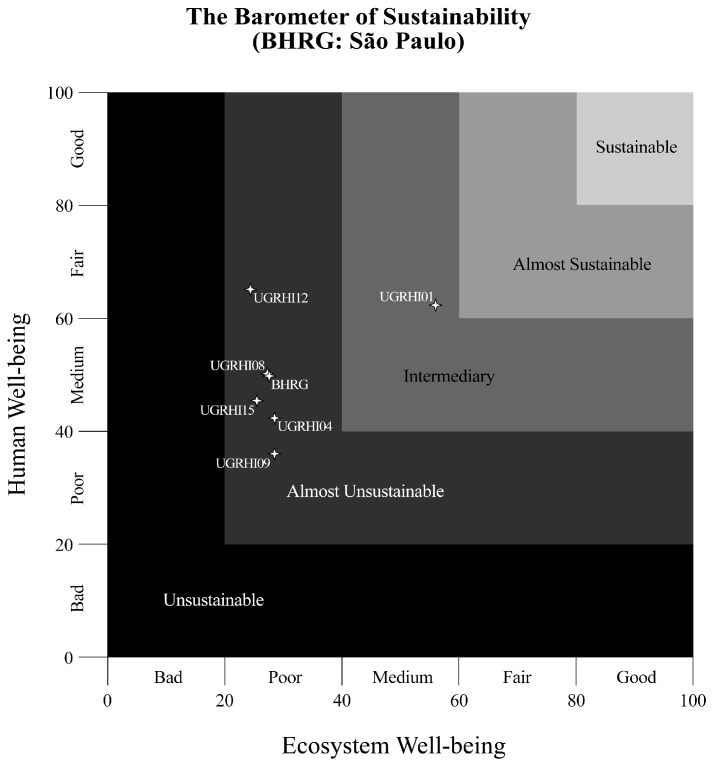
Two-dimensional graphs representing water sustainability condition at BHRG and its respective water management units in São Paulo.

Sustainability analysis tools play an important role in integrating the different sustainable development dimensions, facilitating planning processes, and supporting decision-making. Figure 4 and Figure 5 indicate the two-dimensional BS graph for Minas Gerais and São Paulo, respectively. BHRG was “almost unsustainable”, with a value of 27.63 (“poor”) for environmental well-being and of 49.79 (“average”) for human well-being, with considerable differences between Minas Gerais and São Paulo States.

Concerning human well-being, all water management units in Minas Gerais presented an “average” sustainability condition. Water management units UGRHI01 and UGRHI12 in São Paulo, on the other hand, reached an “acceptable” sustainability condition, and UGRHI09, “poor”. The determinant theme contributing to these results was health, represented by the number of people hospitalized due to waterborne diseases at BHRG (Tables S4 and S5: see indicator number 6).

Concerning environmental well-being, all water management units presented a “poor” condition, except for GD6 and GD7, in Minas Gerais, categorized as a “very bad” sustainability condition. UGRHI01, in São Paulo, was the only water management unit that reached an “intermediate” condition at BHRG. The vegetation theme, represented by native vegetation preservation and conservation units, contributed to these results (Tables S4 and S5: see indicator number 21).

The environmental dimension was decisive for the “almost unsustainable” condition at BHRG, evidencing the imbalance between socioeconomic development and environmental quality. Socioeconomic and water resources development cannot be dissociated from environmental conservation because, in essence, they involve human sustainability in the natural environment [37].

The BS tool was applied at the Jurumirim Basin, Angra dos Reis (Rio de Janeiro—Brazil) and made it possible to evaluate that the watershed fits an intermediate situation in relation to the sustainable development. In contrast to BHRG, it performs better in the environmental subjects than in the socioeconomic ones, being closer to environment conservation than to social equity and economic growth [24]. However, it is important to highlight that the spatial-temporal comparison of the results obtained by BS between different areas is only possible if the system of indicators used in the study and the performance scale of the indicators are the same.

### 3.2. The Relationship between Environment and Human Well-being at BHRG

**Figure 6:**
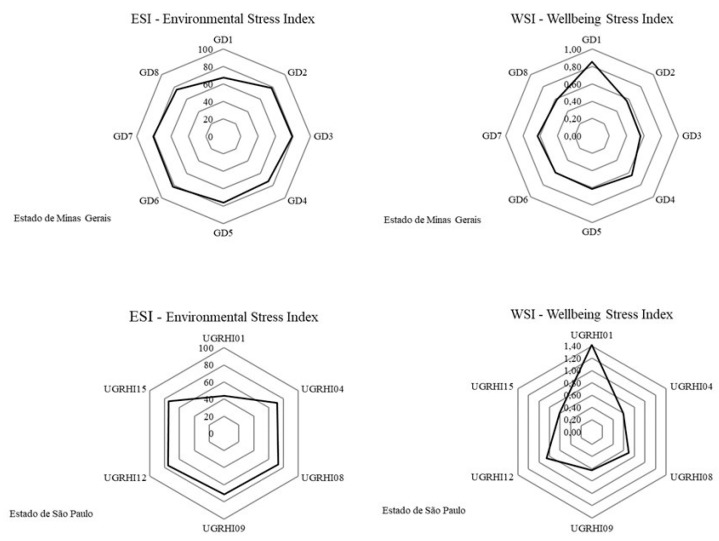
Environmental Stress Index and Well-being Stress Index for the BHRG water management units.

The Environmental Stress Index (ESI) and Well-being Stress Index (WSI) facilitated the understanding of the relationship between human well-being and environmental stress. Figure 6 presents the ESI and WSI values at BRHG for Minas Gerais and São Paulo.

In Minas Gerais, the high ESI percentage values (67.46–81.75%) demonstrated high environmental stress. WSI values lower than 1 (0.56–0.85), allied to high ESI values, demonstrate that socioeconomic development has led to high environmental costs.

The same was observed for São Paulo, where the ESI comprised high environmental stress percentages (71.51–75.56%), with WSI values of less than 1 (0.59–0.86). The exception was UGRHI01, with the lowest environmental stress percentage (43.96%) and a WSI greater than 1 (1.42). Thus, UGRHI01 is the only water management unit that did not cause high environmental stress to maintain human well-being, due to the fact that it presents the highest percentage of preserved native vegetation (51.7%), the smallest population, and is less economically exploited (Table S5).

Less than 15% of the native vegetation is preserved at BHRG (Tables S4 and S5: see indicator number 21). Increased urbanization, agricultural, and industrial activities have augmented the demand for water and energy and consequently led to increased sewage, effluents, and waste generation. However, environmental sanitation and water monitoring were precarious and did not accompany socioeconomic development. In addition, less than 20% of the Rio Grande extension, on average, had its water quality classified from the monitoring (Tables S4 and S5: see indicator numbers 1 and 13). Additionally, the average number of people hospitalized for waterborne diseases was 1.234 in the area (Tables S4 and S5: see indicators number 6).

Despite the reasonable proportion of municipalities connected to water and sewage networks, the proportion of the municipalities displaying landfill and sewage treatment was low (Tables S4 and S5: see indicators numbers 3, 4, 7, 8, 9, 10, and 11). If water and sewage treatment efficiency and landfill quality were to be included in the discussion, the negative impact on water resources would be even greater. In addition, stress and scarcity problems due to global environmental changes and the lack of articulation and consistent actions regarding governance of water resources worsen the water crisis [9]. The consequences are increased sources of contamination, reduced water availability, and difficult access to drinking water, aggravating population health.

### 3.3. Vulnerable Thematic Areas at BHRG

**Figure 7:**
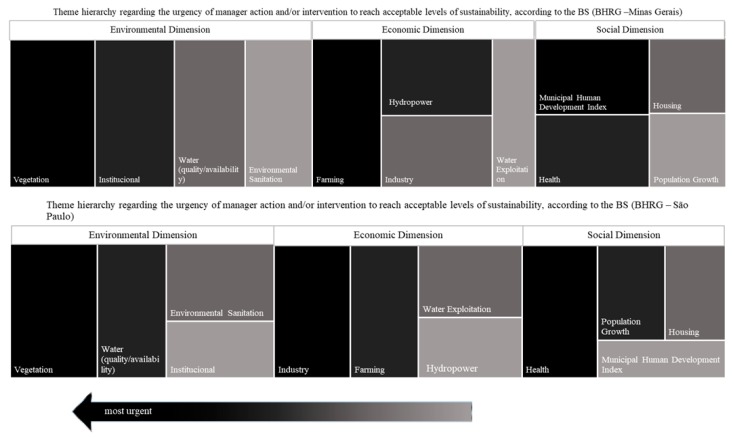
Hierarchy of vulnerable thematic areas at BHRG, separated by sustainable development dimension and by state. The darker tones and the direction of the arrow represent thematic area vulnerability and dimension, respectively.

**Figure 8:**
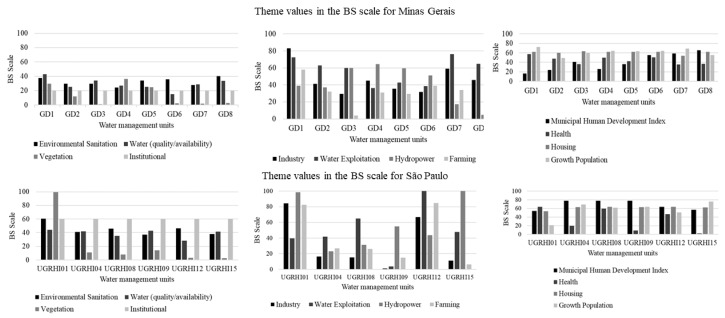
Theme values in the BS scale for the states of Minas Gerais and São Paulo.

The BS application allowed the identification of vulnerable thematic areas, which require greater attention of managers and society. Understanding thematic areas subsidizes action and/or intervention planning, increases water resources management efficiency [10] and provides references for the formulation of public policies [20]. Figure 7 displays the vulnerable thematic areas for Minas Gerais and São Paulo concerning sustainable development dimensions. The mean results of each thematic area were obtained for both states from the theme values in the BS scale (Figure 8).

In Minas Gerais, the thematic areas “vegetation” and “institutional” were the most vulnerable, followed by agriculture, energy generation, Municipal Human Development Index, and health, while “vegetation” and “water quality/quantity” areas were the most vulnerable in São Paulo, followed by “industry”, “agriculture”, “health”, and “population growth”.

Native vegetation recovery at BHRG is essential to reduce environmental stress and improve environmental well-being, but is has been almost completely suppressed, represented by the Mata Atlântica and Cerrado biomes. Vegetation cover type may influence different soil and water attribute behaviors, for example. Native forest removal increases large degradation areas, damaging hydrology and biodiversity. Preserving and/or recovering these areas is essential for the production and conservation of water sources, as they intercept rainwater, provide infiltration conditions and reduce runoff [38,39].

The territorial planning cycle requires greater coordination, cohesion, and integration of sectoral policies. Likewise, it is important to coordinate and integrate other policies in sectors that affect water resources, such as agriculture, industry, forestry, energy, and environmental sanitation, for example [40]. Water policy has developed separately from environmental management, and sectorial water management has fragmented attempts at integrating management [41]. Moreover, a lack of studies on the relationship between these policies makes it difficult to link knowledge on the subject [10]. The need for holistic approaches has been cited several times to understand the complex and interconnected aspects of water governance and management [42].

Improving water management by integrating and optimizing multiple uses, flexibly allocating water, and investing in environmental sanitation is one of the most efficient forms of socioeconomic development, as it leads to improved quality of life, generates jobs and income, and increases the water supply capacity [9].

Thus, it is possible to create an “ideal” BHRG scenario, based on the BS results, considering that:

1. The economic dimension will remain unchanged;
2. The vegetation, environmental sanitation, and water monitoring themes will reach the minimum values for an “acceptable” sustainability condition on the BS scale (value = 61);
3. The institutional theme will reach the maximum value in the BS scale (value = 100) and
4. The housing theme, belonging to the social dimension, will reach the minimum acceptable value in the BS scale (value = 61).

**Figure 9:**
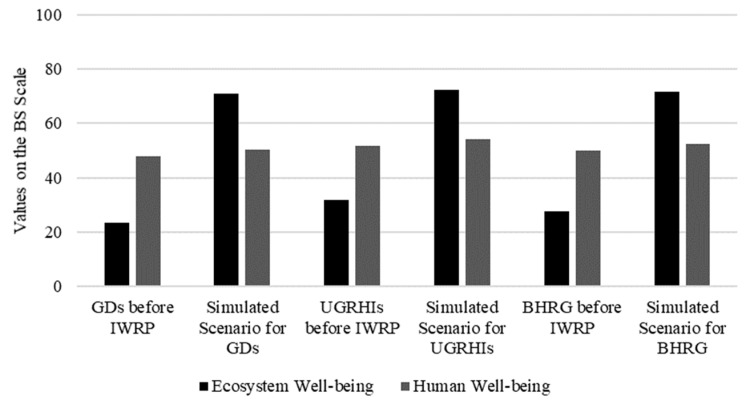
GDs (Minas Gerais Water Management Units), UGRHIs (São Paulo Water Management Units), and IWRP (Integrated Water Resources Plan). Scenario created for the BHRG with the economic dimension unchanged. Changes in the social dimension (housing thematic area) and the environmental dimension (all thematic areas) were considered. This scenario consists of implementing all water management instruments and achieving an “acceptable” condition for environmental sanitation, water monitoring, native vegetation, and housing. The mean values for all water management units (on the BS scale) were used.

The simulation for this scenario is exhibited in Figure 9. The results indicate that, if the described changes occur, BHRG will present acceptable sustainability conditions (values in the BS scale > 60). Thus, it is possible to state that BHRG water resources will reach an “acceptable” level of sustainability only if a reduction in environmental stress occurs. Ignoring the consequences of a focus on economic efficiency can drive policies that undermine the conditions of the poor. Sustainable water governance comprehends the justice of policies and actions and their impacts on all stakeholders [42] (p. 5).

The BS integrated the social, environmental, and economic dimensions, identifying the vulnerable thematic areas in the assessed basin, being able to reference public policies, assist in planning, and increase the effectiveness of water resource management.

The results of this study may differ when applying other sustainability analysis methods or another indicator system. First, BS is flexible concerning the choice of indicators. Second, the performance scale elaborated by the analyst may be variable. Nevertheless, flexibility and transparency are the major advantages of BS. When the sustainability analysis method presents a fixed indicator system, the analyst is totally dependent on the availability of specific data. That is, if an indicator is unavailable for the study area, the calculation will be unfeasible. The subjectivity in the creation of the BS performance scale may interfere in the sustainability condition of the study area. Thus, the use of indicators and dimension-weighting methods can be viable alternatives. Some limitations concerning the availability of some indicators considered relevant for the sustainability condition analysis of the BHRG water resources. Soil erosion indicators, for example, were not considered in this study, as there is no data available for the State of Minas Gerais. Despite this, the study presented results consistent with the reality of the BHRG when compared to the diagnosis of the situation of the water resources of the basin. In addition, it has also contributed to the integration of social, economic, and environmental variables under the sustainability approach.

This study makes an important contribution, as the system of indicators used here was created from official BHRG data. This allows any other river basins to replicate the methodology from the official data of each area, respecting local peculiarities and data availability. For BHRG, specifically, this study can aid in the monitoring of the IWRP, since the applied indicators are often made available by Brazilian government agencies and, therefore, were used in the diagnosis of the water resources situation of the BHRG. In addition, the performance scales were constructed from the goals and objectives established by the BHRG committees themselves, as well as the scientific literature and current legislation.

Indicators of sustainable development should be selected and negotiated by the appropriate communities of interest. Thus, a composite indicator must be constructed within a coherent framework. This would ensure that the specific parameters involved in the evaluation process could change through time according to the interests of the particular stakeholders involved in the construction of the indicator [43]. A reapplication of the BS after the IWRP implementation at BHRG is recommended, to monitor the area and carry out spatio-temporal comparisons.

Overall, identifying the sustainability condition of watersheds by applying the BS has contributed to a greater understanding of the relationships that exist between humans and the environment and provided reference points for policy formulations and water management.

## 4. Conclusions

This study explored using the Barometer of Sustainability (BS) tool to identify the water sustainability condition at the Rio Grande Basin (BHRG), before the implementation of Integrated Water Resources Plan (IWRP) and to identify vulnerable thematic areas that require more attention by managers and society.

The water condition at the River Grande Basin was “almost unsustainable”, with a considerable difference between Minas Gerais and São Paulo. However, ecosystem and human well-being presented “almost unsustainable” and “intermediary” conditions, respectively, in both states.

The Environmental Stress Index (ESI) and Well-being Stress Index (WSI) confirm the BS results, demonstrating the high environmental stress concerning human well-being maintenance at BHRG. UGRHI 01, a water management unit located in São Paulo, was the only one in an intermediate “sustainability” condition.

The imbalance between socioeconomic development and environmental quality at BHRG is evident. Urban growth, farming, industrial activities, and the high hydroelectric potential installed in the river basin contributed to this imbalance. Moreover, an extensive suppressed native vegetation area, low effectiveness of water policies, poor environmental sanitation, and inefficient water monitoring system also contribute. This imbalance degrades water and affects public health. Native vegetation recovery requires more attention by managers and society among the most vulnerable thematic areas, in both states, as native vegetation plays an important role in soil and water conservation and, consequently, in the water quality maintenance and availability.

The BS is a flexible and easy-to-apply tool, since it integrates sustainable development dimensions, identifies vulnerable thematic areas, and displays the results in a didactic form, significantly useful for managers, as it facilitates decision making.

Given the importance of sustainability studies, it may be interesting to reapply the BS after the IWRP implementation at BHRG. When elaborating the performance scale, the indicators can be weighted to reduce analysis tool subjectivity. Moreover, the BS also displays application potential in other areas and scales for spatio-temporal monitoring and comparison purposes.

In general, identifying the sustainability condition in watersheds contributes to knowledge on relationships that exist between humans and the environment and provides reference points for policy formulations and water management. The BS approach was efficient for this study, although it presents certain limitations.

## Supplementary Materials

The following are available online at http://www.mdpi.com/1660-4601/15/11/2582/s1, Table S1: Reference and description of environmental dimension sustainability indicators separated by theme, Table S2: Reference and description of economic dimension sustainability indicators separated by theme, Table S3: Reference and description of social dimension sustainability indicators separated by theme, Table S4: The actual sustainability indicator values selected for the River Grande Basin—Minas Gerais, Table S5: The actual sustainability development indicator values selected for the River Grande Basin—São Paulo, Table S6: Performance scale of sustainable development indicators associated to the sustainability barometer scale.
