# Supplementary material for: Water Sustainability at the River Grande Basin, Brazil: An Approach Based on the Barometer of Sustainability
Source: Int J Environ Res Public Health. 2018 Nov 19;15(11):2582. doi: 10.3390/ijerph15112582 (PMC6266740; doi:10.3390/ijerph15112582)
Supplement: Supplementary file 1 [file ijerph-15-02582-s001.zip › ijerph-382245 - supplementary proofreading revised/SUP_1.docx]

Supplementary Materials

Water Sustainability at the River Grande Basin, Brazil: An Approach Based on the Barometer of Sustainability

Janaína Ferreira Guidolini, Angélica Giarolla, Peter Mann Toledo, Carlos Alberto Valera and Jean Pierre Henry Balbaud Ometto

**Table S1.** Reference and description of environmental dimension sustainability indicators separated by theme

| **Environmental Dimension** | | |
| --- | --- | --- |
| **Indicator** | **Reference** | **Description** |
| Theme 1: Environmental Sanitation | | |
| Estimated quantity of sewage produced per year (m³/year) | Quantity of sewage produced < 5 was considered sustainable (IPT, 2008). | It was considered the IBGE (Brazilian Institute of Geography and Statistics) census of 2000s. It also considered that the average water consumption was (175 liters/ inhabitant/day), and the water/water ratio was 0.85 and 365 days a year. |
| Estimated amount of domestic solid waste produced per year (ton/year) | Amount of solid waste produced < 5 was considered sustainable (IPT, 2008). | It was considered the 2000s IBGE (Brazilian Institute of Geography and Statistics) census and the average production of solid wastes of 0.80 kg/ inhabitants/day and 365 days per year. |
| Proportion of municipalities with sewage treatment in ETE (Sewage Treatment Plant) (%) | Goal established for this work: 100 | Indicator that reflects the municipalities with sewage treatment in ETE. |
| Proportion of municipalities with landfill (%) | Goal established for this work: 100 | The indicator reflects the municipalities that destine the solid waste properly, in landfills. |
| Proportion of municipalities with 100% of households with garbage collection. | Goal established for this work: 100 | Indicator reflects municipalities that have garbage collection. |
| Theme 2: Water (quality/availability) | | |
| Proportion of water courses monitored and classified as optimal/excellent/good (%). | It was considered sustainable when > 90% of the monitored water courses had an optimal/excellent/good rating (IPT, 2008). | The indicator reflects the water quality, obtained from the IQA (Water Quality Index). |
| Proportion of the waterways extents classified by means of monitoring (%). | It was considered sustainable > 60% (IPT, 2008). | Comprehensiveness of surface water quality monitoring. |
| Number of fluviometers installed (nº). | Fluviometers assist in monitoring the flow of water courses. Without knowing the amount of water available for use, management is hampered. So, it was adopted as sustainable above 16 fluviometers installed (IPT, 2008). | This indicator reflects the monitoring of the water courses flow. |
| Number of wells monitored (nº) | Comprehensiveness of groundwater monitoring. The higher the monitoring, better the knowledge about the groundwater quality. Above 16 wells was considered sustainable (IPT, 2008). | Comprehensiveness of groundwater quality monitoring. |
| Estimated amount of treated water consumed per year (m³/year). | It was considered sustainable value < 10 x 10^6^ (IPT, 2008). | The indicator is expressed in the estimated amount of treated water required for human consumption per year. It is obtained by multiplying the daily per capita consumption of water (estimated), by the number of days in the year and by the total number of inhabitants. |
| Theme 3: Vegetation | | |
| The proportion of protected areas by Conservation Units (%). | Conservation Units (UCs) are important for maintaining biodiversity and soil and water conservation. For this work, were considered sustainable to be > 50% protected by UCs (IPT, 2008). | The proportion of the areas protected by UCs. Important protection for the maintenance of biodiversity, conservation of soil and water resources. |
| Proportion of area with native vegetation (%). | We consider above 50% sustainable (IPT, 2008). | The area with native vegetation cover of arboreal size. An important indicator reflects how much still exists of native vegetation, with special attention to the areas of permanent preservation. |
| Theme 4: Institutional | | |
| Number of NWRP (National Water Resources Policy) instruments implemented (nº). | Goal established for this work: 5 | The instruments of the NWRP are: (1) water resources plan, (2) framing of water bodies; (3) granting the right to use water resources, (4) charging for the use of water resources, and (5) information system on water resources. Compensation to municipalities, although it continues to appear as an instrument of the PNRH, had its provisions vetoed in the text of the PNRH itself. Therefore, this instrument was not considered in this study. |
