# Supplementary material for: Water Sustainability at the River Grande Basin, Brazil: An Approach Based on the Barometer of Sustainability
Source: Int J Environ Res Public Health. 2018 Nov 19;15(11):2582. doi: 10.3390/ijerph15112582 (PMC6266740; doi:10.3390/ijerph15112582)
Supplement: Supplementary file 1 [file ijerph-15-02582-s001.zip › ijerph-382245 - supplementary proofreading revised/SUP_2.docx]

Supplementary Materials

Water Sustainability at the River Grande Basin, Brazil: An Approach Based on the Barometer of Sustainability

Janaína Ferreira Guidolini, Angélica Giarolla, Peter Mann Toledo, Carlos Alberto Valera and Jean Pierre Balbaud Ometto

**Table S2.** Reference and description of economic dimension sustainability indicators separated by theme

| **Economic Dimension** | | |
| --- | --- | --- |
| **Indicator** | **Reference** | **Description** |
| Theme 5: Industry | | |
| Number of industrial establishments (nº.). | Quantity > 3000 was considered unsustainable (IPT, 2008). | The indicator reflects the number of industries in the BHRG (River Grande Basin). Industries move the economy, but they put a lot of pressure on water resources. Moreover, they are potential generators of solid waste, liquid effluents contaminating water and soil and air polluters. |
| Theme 6: Water Exploitation | | |
| Quantity of mining operations of mineral water (nº). | Amount < 10 was considered sustainable (IPT, 2008). | Exploitation of mineral water and drinking water table. |
| Theme 7: Farming | | |
| Number of agricultural establishments (nº). | Quantity > 4000 was considered unsustainable (IPT, 2008). | Indicator that reflects the number of agricultural establishments in the BHRG. Most of the Basin area is used for agricultural purposes. Farming drives the economy, but it puts a lot of pressure on water resources, requiring a large amount of water for production. It can also negatively impact, with excessive soil losses, diffuse pollution, contamination due to excess fertilizers, agrochemicals, etc. |
| Theme 8: Hydropower | | |
| Hydropower Capacity installed (kW) | Capacity > 1 million was considered unsustainable (IPT, 2008) | The indicator reflects the capacity of hydroelectric generation in the BHRG. It moves the economy but requires integrated, participative and shared management to manage the conflicts that arise due to the implantation and operation of the plants, not to hurt the consumptive water use. |
