# Supplementary material for: Water Sustainability at the River Grande Basin, Brazil: An Approach Based on the Barometer of Sustainability
Source: Int J Environ Res Public Health. 2018 Nov 19;15(11):2582. doi: 10.3390/ijerph15112582 (PMC6266740; doi:10.3390/ijerph15112582)
Supplement: Supplementary file 1 [file ijerph-15-02582-s001.zip › ijerph-382245 - supplementary proofreading revised/SUP_3.docx]

Supplementary Materials

Water Sustainability at the River Grande Basin, Brazil: An Approach Based on the Barometer of Sustainability

Janaína Ferreira Guidolini, Angélica Giarolla, Peter Mann Toledo, Carlos Alberto Valera and Jean Pierre Henry Balbaud Ometto

**Table S3.** Reference and description social dimension sustainability indicators separated by theme

| **Social Dimension** | | |
| --- | --- | --- |
| **Indicator** | **Reference** | **Description** |
| Theme 9: Human Development | | |
| Municipal Human Development Index - MHDI (dimensionless) | In this study, values > 0.800 of the MHDI were considered sustainable (IPT, 2008). | The indicator considered the longevity, income, and education. |
| Theme 10: Health | | |
| Annual number of records for hospitalization for waterborne diseases (nº). | Goal established for this work: 0 | The indicator reflects the number of people who were hospitalized due to ingestion of water of inadequate quality. The following diseases were considered: cholera; typhoid and paratyphoid fevers; amoebiasis; acute diarrheal diseases (diarrhea and gastroenteritis of presumed infectious origin and other infectious intestinal diseases); leptospirosis and hepatitis A and E. |
| Theme 11: Housing | | |
| Proportion of municipalities connected to the water supply network (%) | Goal established for this work: 100% | The indicator reflects the access to the water supply network of the municipalities of the River Grande Basin (BHRG). |
| Proportion of households connected to the sewerage network (%) | Goal established for this work: 100% | The indicator reflects the access to sewage network of the municipalities of the BHRG. |
| Theme 12: Population | | |
| Geometric annual growth rate (% per year) | Accelerated population growth makes it difficult to achieve sustainability. Thus, rates > 1.8 were considered unsustainable (IPT, 2008). | Indicator expresses population growth. |
