# Supplementary material for: Water Sustainability at the River Grande Basin, Brazil: An Approach Based on the Barometer of Sustainability
Source: Int J Environ Res Public Health. 2018 Nov 19;15(11):2582. doi: 10.3390/ijerph15112582 (PMC6266740; doi:10.3390/ijerph15112582)
Supplement: Supplementary file 1 [file ijerph-15-02582-s001.zip › ijerph-382245 - supplementary proofreading revised/SUP_4.docx]

Supplementary Materials

Water Sustainability at the River Grande Basin, Brazil: An Approach Based on the Barometer of Sustainability

Janaína Ferreira Guidolini, Angélica Giarolla, Peter Mann Toledo, Carlos Alberto Valera and Jean Pierre Henry Balbaud Ometto

**Table S4.** The actual sustainability indicators values selected for the River Grande Basin–Minas Gerais

| **Indicator** | **GD1** | **GD2** | **GD3** | **GD4** | **GD5** | **GD6** | **GD7** | **GD8** |  |
| --- | --- | --- | --- | --- | --- | --- | --- | --- | --- |
| 1 Proportion of the waterways extents classified by means of monitoring | 50.72 | 14.51 | 99.97 | 24.94 | 36.37 | 0 | 52.84 | 76.38 |  |
| 2 Number of industrial establishments | 177 | 1987 | 2552 | 1779 | 2231 | 2436 | 1037 | 1749 |  |
| 3 Estimated quantity of sewage produced per year | 5.58 | 27.62 | 38.12 | 23.05 | 28.41 | 20.89 | 15.91 | 24.11 |  |
| 4 Estimated amount of domestic solid waste produced per year | 29.98 | 148.56 | 205.01 | 123.99 | 152.77 | 112.36 | 85.59 | 129.67 |  |
| 5 Municipal Human Development Index | 0.733 | 0.743 | 0.78 | 0.765 | 0.756 | 0.775 | 0.779 | 0.785 |  |
| 6 Annual number of records for hospitalization for waterborne diseases | 224 | 677 | 1183 | 586 | 945 | 556 | 1220 | 1176 |  |
| 7 Proportion of municipalities connected to the water supply network | 97.32 | 95.44 | 98.62 | 97.34 | 97.46 | 97.82 | 99.45 | 97.47 |  |
| 8 Proportion of households connected to the sewerage network | 76.95 | 76.43 | 88.31 | 91.45 | 91.28 | 91.61 | 93.78 | 84.65 |  |
| 9 Proportion of municipalities with sewage treatment in ETE (Sewage Treatment Plant | 4.76 | 6.9 | 16.67 | 8.7 | 0 | 4.76 | 5.56 | 22.22 |  |
| 10 Proportion of municipalities with 100% of households with garbage collection. | 28.57 | 41.38 | 52.78 | 21.74 | 71.79 | 71.43 | 16.67 | 77.78 |  |
| 11 Proportion of municipalities with landfill | 9.52 | 10.34 | 16.67 | 17.39 | 20.51 | 19.05 | 33.33 | 38.89 |  |
| 12 Number of wells monitored | 0 | 18.8 | 0 | 0 | 0 | 0 | 0 | 0 |  |
| 13 Proportion of water courses monitored and classified as optimal/excellent/good | 28.12 | 0 | 9.76 | 33.57 | 20.22 | 4.6 | 14.17 | 9.52 |  |
| 14 Proportion of protected areas by Conservation Units | 13.29 | 1 | 0 | 23.82 | 6.33 | 0 | 0.04 | 0 |  |
| 15 Number of NWRP (National Water Resources Policy) instruments implemented | 1 | 1 | 1 | 2 | 1 | 1 | 1 | 1 |  |
| 16 Geometric annual growth rate (TGCA) | 0.34 | 1.34 | 1.35 | 1.67 | 1.5 | 1.52 | 1.47 | 1.12 |  |
| 17 Quantity of mining operations of mineral water | 14 | 19 | 21 | 52 | 38 | 45 | 12 | 18 |  |
| 18 Hydropower Capacity installed | 143220 | 244640 | 10190 | 9100 | 11780 | 51320 | 1705200 | 4833200 |  |
| 19 Estimated amount of treated water consumed per year | 6560000 | 32500000 | 44850000 | 27120000 | 33420000 |  | 18720000 | 28360000 |  |
| 20 Number of fluviometers installed | 4 | 1 | 0 | 0 | 0 |  | 0 | 1 |  |
| 21 Proportion of the area with native vegetation | 7.18 | 1.55 | 0.98 | 6.85 | 8.65 |  | 1.26 | 2.76 |  |
| 22 Number of agricultural establishments | 1114 | 2813 | 5977 | 2938 | 3116 |  | 2615 | 4385 |  |
