# Supplementary material for: Water Sustainability at the River Grande Basin, Brazil: An Approach Based on the Barometer of Sustainability
Source: Int J Environ Res Public Health. 2018 Nov 19;15(11):2582. doi: 10.3390/ijerph15112582 (PMC6266740; doi:10.3390/ijerph15112582)
Supplement: Supplementary file 1 [file ijerph-15-02582-s001.zip › ijerph-382245 - supplementary proofreading revised/SUP_5.docx]

Supplementary Materials

Water Sustainability at the River Grande Basin, Brazil: An Approach Based on the Barometer of Sustainability

Janaína Ferreira Guidolini, Angélica Giarolla, Peter Mann Toledo, Carlos Alberto Valera and Jean Pierre Henry Balbaud Ometto

**Table S5.** The actual sustainability indicators values selected for the River Grande Basin–São Paulo

| **Indicator** | **UGRHI01** | **UGRHI04** | **UGRHI08** | **UGRHI09** | **UGRHI12** | **UGRHI15** |
| --- | --- | --- | --- | --- | --- | --- |
| 1 Proportion of the waterways extents classified by means of monitoring | 78.55 | 77.79 | 21.68 | 75.56 | 72.12 | 70.88 |
| 2 Number of industrial establishments | 165 | 3338 | 3471 | 4891 | 725 | 3889 |
| 3 Estimated quantity of sewage produced per year | 3.32 | 52.86 | 33.22 | 71.67 | 16.99 | 59.61 |
| 4 Estimated amount of domestic solid waste produced per year | 17.84 | 284.27 | 178.64 | 385.47 | 91.37 | 320.58 |
| 5 Municipal Human Development Index (MHDI) | 0.774 | 0.798 | 0.798 | 0.798 | 0.783 | 0.777 |
| 6 Annual number of records for hospitalization for waterborne diseases | 85 | 2040 | 1001 | 3085 | 736 | 3768 |
| 7 Proportion of municipalities connected to the water supply network | 88.79 | 98.54 | 99.12 | 98.11 | 98.94 | 97.73 |
| 8 Proportion of households connected to the sewerage network | 50.03 | 93.95 | 90.37 | 91.37 | 80.12 | 97.08 |
| 9 Proportion of municipalities with sewage treatment in ETE (Sewage Treatment Plant | 9.58 | 44.43 | 72.6 | 37.83 | 44.54 | 21.56 |
| 10 Proportion of municipalities with 100% of households with garbage collection. | 100 | 91.3 | 59.1 | 89.47 | 75 | 90.62 |
| 11 Proportion of municipalities with landfill | 66.67 | 30.43 | 50 | 50 | 50 | 50 |
| 12 Number of wells monitored | 0 | 11 | 5 | 12 | 3 | 14 |
| 13 Proportion of water courses monitored and classified as optimal/excellent/good | 35.47 | 15.31 | 17.56 | 10.96 | 8.4 | 16.52 |
| 14 Proportion of protected areas by Conservation Units | 100 | 0.66 | 0.4 | 1.53 | 0.01 | 0.08 |
| 15 Number of NWRP (National Water Resources Policy) instruments implemented | 3 | 3 | 3 | 3 | 3 | 3 |
| 16 Geometric annual growth rate (GAGR) | 1.96 | 1.67 | 1.9 | 2 | 1.21 | 1.65 |
| 17 Quantity of mining operations of mineral water | 41 | 39 | 18 | 346 | 8 | 33 |
| 18 Hydropower Capacity installed | 360 | 250980 | 503470 | 33910 | 0 | 0 |
| 19 Estimated amount of treated water consumed per year | 3900000 | 62180000 | 39080000 | 1577010000 | 19990000 | 70130000 |
| 20 Number of fluviometers installed | 1 | 7 | 11 | 10 | 4 | 8 |
| 21 Proportion of the area with native vegetation | 51.17 | 3.78 | 2.47 | 2.95 | 3.05 | 1.87 |
| 22 Number of agricultural establishments | 92 | 3396 | 3457 | 4649 | 1860 | 5690 |
