# Supplementary material for: Water Sustainability at the River Grande Basin, Brazil: An Approach Based on the Barometer of Sustainability
Source: Int J Environ Res Public Health. 2018 Nov 19;15(11):2582. doi: 10.3390/ijerph15112582 (PMC6266740; doi:10.3390/ijerph15112582)
Supplement: Supplementary file 1 [file ijerph-15-02582-s001.zip › ijerph-382245 - supplementary proofreading revised/SUP_6.docx]

Supplementary Materials

Water Sustainability at the River Grande Basin, Brazil: An Approach Based on the Barometer of Sustainability

Janaína Ferreira Guidolini, Angélica Giarolla, Peter Mann Toledo, Carlos Alberto Valera and Jean Pierre Henry Balbaud Ometto

**Table S6.** Performance scale of sustainable development indicators associated to the sustainability barometer scale

| **Sustainable Development Indicators** | **Barometer of Sustainability Scale** | | | | |
| --- | --- | --- | --- | --- | --- |
|  | **0 ≤ 20** | **20 ≤ 40** | **40 ≤ 60** | **60 ≤ 80** | **80 ≤ 100** |
|  | **Unsustainable** | **Almost Unsustainable** | **Intermediary** | **Almost Sustainable** | **Sustainable** |
|  | **Indicator Performance Scale** | | | | |
| Proportion of water courses monitored and classified as optimal/excellent/good | <30 | 30–50 | 51–70 | 71–90 | >90 |
| Number of fluviometers installed | <3 | 3–5 | 6–10 | 11–16 | >16 |
| Estimated amount of treated water consumed per year | >80.10 ^6^ | 60.10 ^6^–80.10 ^6^ | 30.10 ^6^–60.10 ^6^ | 10.10 ^6^–30.10 ^6^ | <10.10 ^6^ |
| Proportion of protected areas by Conservation Units | 0 | 1–10 | 11–25 | 25–50 | >50 |
| Proportion of the area with native vegetation | <10 | 10–19 | 20–39 | 40–49 | >50 |
| Estimated quantity of sewage produced per year | >60 | 31–60 | 21–30 | 5–20 | <5 |
| Estimated amount of domestic solid waste produced per year | >350 | 201–300 | 51–200 | 5–50 | <5 |
| Geometric annual growth rate | >1.80 | 1.61–1.80 | 1.21–1.60 | 0.40–1.20 | <0.40 |
| Municipal Human Development Index (MHDI) | <0.740 | 0.740–0.759 | 0.760–0.779 | 0.780–0.800 | <0.800 |
| Proportion of the waterways extents classified by means of monitoring | <10 | 10–20 | 21–40 | 41–60 | >60 |
| Number of wells monitored | <3 | 3–5 | 6–10 | 11–16 | >16 |
| Proportion of municipalities with sewage treatment in ETE (Sewage Treatment Plant | 0–74 | 75–84 | 85–94 | 95–99 | 100 |
| Proportion of municipalities with 100% of households with garbage collection. | <75 | 75–84 | 85–94 | 95–99 | 100 |
| Proportion of municipalities with landfill (%) | <75 | 75–84 | 85–94 | 95–99 | 100 |
| Proportion of municipalities connected to the water supply network (%) | <75 | 75–84 | 85–94 | 95–99 | 100 |
| Annual number of records for hospitalization for waterborne diseases (nº) | >2000 | 1001–2000 | 100–1000 | 1–100 | 0 |
| Number of NWRP (National Water Resources Policy) instruments implemented (nº) | 0–1 | 2 | 3 | 4 | 5 |
| Number of agricultural establishments (nº) | >4000 | 2001–4000 | 1001–2000 | 100–1000 | <100 |
| Quantity of mining operations of mineral water (nº) | >100 | 40–100 | 20–39 | 10–19 | <10 |
| Number of industrial establishments (nº) | >3000 | 2001–3000 | 1001–2000 | 200–1000 | <200 |
| Hydropower Capacity installed (kW) | >1 mi | 100mil–1mi | 11mil–99.9 mil | 1–10.9 mil | 0–9.9 mil |
